# Streptococcal H_2_O_2_ inhibits IgE-triggered degranulation of RBL-2H3 mast cell/basophil cell line by inducing cell death

**DOI:** 10.1371/journal.pone.0231101

**Published:** 2020-04-17

**Authors:** Nobuo Okahashi, Masanobu Nakata, Yujiro Hirose, Hirobumi Morisaki, Hideo Kataoka, Hirotaka Kuwata, Shigetada Kawabata

**Affiliations:** 1 Center for Frontier Oral Science, Osaka University Graduate School of Dentistry, Suita, Osaka, Japan; 2 Department of Oral and Molecular Microbiology, Osaka University Graduate School of Dentistry, Suita, Osaka, Japan; 3 Department of Oral Microbiology and Immunology, School of Dentistry, Showa University, Shinagawa, Tokyo, Japan; 4 Department of Oral Microbiology, Asahi University School of Dentistry, Mizuho, Gifu, Japan; King's College London, UNITED KINGDOM

## Abstract

Mast cells and basophils are central players in allergic reactions triggered by immunoglobulin E (IgE). They have intracellular granules containing allergic mediators (e.g., histamine, serotonin, inflammatory cytokines, proteases and β-hexosaminidase), and stimulation by IgE-allergen complex leads to the release of such allergic mediators from the granules, that is, degranulation. Mast cells are residents of mucosal surfaces, including those of nasal and oral cavities, and play an important role in the innate defense system. Members of the mitis group streptococci such as *Streptococcus oralis*, are primary colonizers of the human oral cavity. They produce hydrogen peroxide (H_2_O_2_) as a by-product of sugar metabolism. In this study, we investigated the effects of streptococcal infection on RBL-2H3 mast cell/basophil cell line. Infection by oral streptococci did not induce degranulation of the cells. Stimulation of the RBL-2H3 cells with anti-dinitrophenol (DNP) IgE and DNP-conjugated human serum albumin triggers degranulation with the release of β-hexosaminidase. We found that *S*. *oralis* and other mitis group streptococci inhibited the IgE-triggered degranulation of RBL-2H3 cells. Since mitis group streptococci produce H_2_O_2_, we examined the effect of *S*. *oralis* mutant strain deficient in producing H_2_O_2,_ and found that they lost the ability to suppress the degranulation. Moreover, H_2_O_2_ alone inhibited the IgE-induced degranulation. Subsequent analysis suggested that the inhibition of degranulation was related to the cytotoxicity of streptococcal H_2_O_2_. Activated RBL-2H3 cells produce interleukin-4 (IL-4); however, IL-4 production was not induced by streptococcal H_2_O_2_. Furthermore, an *in vivo* study using the murine pollen-induced allergic rhinitis model suggested that the streptococcal H_2_O_2_ reduces nasal allergic reaction. These findings reveal that H_2_O_2_ produced by oral mitis group streptococci inhibits IgE-stimulated degranulation by inducing cell death. Consequently, streptococcal H_2_O_2_ can be considered to modulate the allergic reaction in mucosal surfaces.

## Introduction

*Streptococcus oralis*, *Streptococcus sanguinis*, and *Streptococcus gordonii* are oral mitis group streptococci, which are the most abundant inhabitants of the oral cavity and dental plaque [1, 2, 3, 4, 5]. They cause a variety of infectious complications such as bacteremia and infective endocarditis [5, 6, 7, 8, 9]. *Streptococcus pneumoniae*, an important pathogen that causes pneumonia, also belongs to the mitis group [8]. These mitis group streptococci produce hydrogen peroxide (H_2_O_2_) as a by-product of sugar metabolism [1, 3, 8, 10, 11, 12, 13, 14].

Mast cells and basophils are key effector cells in immunoglobulin E (IgE)-associated immune response, for example, anaphylaxis and allergic disorders such as allergic rhinitis and pollen-induced allergic rhinitis, that is, pollinosis [15, 16, 17, 18]. They have intracellular granules containing allergic mediators (e.g., histamine, serotonin, inflammatory cytokines, proteases and β-hexosaminidase) [15, 16, 18]. These cells constitutively express the IgE receptor (FcεRI) on their surface, and its aggregation by the IgE-allergen complex eventually leads to the release of such allergic mediators from the granules, that is, degranulation [15, 16, 17, 18]. In addition, they are associated with the innate immune response as well as autoimmune diseases, and also contribute to the initiation and progression of oral pathological conditions [15, 16, 18, 19, 20, 21]. Recently, as an allergen immunotherapy, sublingual immunotherapy has become a common treatment for pollinosis [22]. However, the influence of oral bacteria on mast cells in oral tissues and sublingual immunotherapy is unclear.

Previous studies [23, 24] have shown that infection with *S*. *pneumoniae* can activate mast cells. Other studies have reported that streptococcal toxins such as the pyrogenic exotoxin of *Streptococcus pyogenes* and hemolytic lipid toxin of *Streptococcus agalactiae* stimulate the degranulation of mast cells [25, 26]. These studies also suggest that modulation of mast cell function may contribute to the infection or colonization of the pathogenic streptococci.

We had previously reported that H_2_O_2_ produced by the oral mitis group streptococci induces the cell death of macrophages, epithelial cells and neutrophils, and its cytotoxicity is likely to contribute to the evasion of the streptococci from the host defense system [14, 27, 28, 29, 30]. Although our previous studies showed that streptococcal H_2_O_2_ is cytotoxic, the unique immune response of mast cells and basophils, i.e., IgE-induced degranulation, would raise another question. In this study, we investigated whether H_2_O_2_ produced by the oral mitis group streptococci is implicated in the allergic function.

## Materials and methods

### Ethics statement

The mouse experiments were performed with the approval of the animal care committee of the Osaka University Graduate School of Dentistry (No, 29-009-0). All experiments were performed according to the guidelines for animal treatment of the committee.

### Chemicals and reagents

Brain heart infusion (BHI) broth was purchased from Becton Dickinson (Sparks, MD, USA). Dulbecco’s modified Eagle’s medium (DMEM) and other cell culture reagents were purchased from Invitrogen (Carlsbad, CA, USA). Mouse anti-dinitrophenol (DNP) IgE monoclonal antibody, DNP-conjugated human serum albumin (HSA), *p*-nitrophenyl-*N*-acetyl-β-d-glucosaminide (PNAG), phorbol 12-myristate 13-acetate (PMA), ionomycin, trypan blue, staurosporine, and catalase were obtained from Sigma Aldrich (St. Louis, MO, USA). Astra Blue dye solution was purchased from ScyTek (Logan UT, USA). LysoTracker Red and SYBR Green II were purchased from Molecular Probes (Eugene, OR, USA) and TaKaRa Bio (Otsu, Japan), respectively. 4,6-Diamidino-2-phenylindole (DAPI) and AlexaFluor 594-conjugated phalloidin were obtained from Dojindo Molecular Technologies (Kumamoto, Japan) and Molecular Probes, respectively. Rabbit anti-β-actin antibody and horseradish peroxidase (HRP)-conjugated anti-rabbit secondary antibody were purchased from Cell Signaling (Danvers, MA, USA). Other commonly used reagents were purchased from Nakalai Tesque (Kyoto, Japan) and Sigma-Aldrich.

### Bacterial strains and culture conditions

*S*. *oralis* ATCC 35037, a type strain originally isolated from the human mouth [2], was obtained from the Japan Collection of Microorganisms at the RIKEN BioResource Center (Tsukuba, Japan). The *spxB*-deletion mutant, *spxB* KO (deficient for H_2_O_2_ production), was generated from *S*. *oralis* ATCC 35037 wild type (WT), as described previously [14].

*Streptococcus salivarius* HHT and *S*. *gordonii* ATCC 10558 were selected from the stock culture collection at the Department of Oral and Molecular Microbiology, Osaka University Graduate School of Dentistry (Osaka, Japan). *S*. *salivarius* does not produce detectable H_2_O_2_ [1, 3], and *S*. *gordonii* is a member of the oral mitis group of streptococci [3, 10, 13]. These bacteria were cultured in BHI broth.

### Cell culture

The rat mast cell/basophil cell line RBL-2H3 (JCRB0023) [31] was obtained from the JCRB Cell Bank (Ibaraki-Osaka, Japan). The cell line has been widely used as a mast cell line in the IgE-stimulated degranulation studies, however, recent studies suggested that this cell line share some characteristics with basophils [32, 33]. The cells were cultured in DMEM supplemented with 5% fetal bovine serum (FBS), penicillin (100 U/mL), and streptomycin (100 μg/mL) at 37°C in a 5% CO_2_ atmosphere. For the degranulation assay (see below), the cells were cultured in 5% FBS DMEM containing no phenol red.

### Effects of streptococcal infection and H_2_O_2_ on degranulation of RBL-2H3 cells

The RBL-2H3 cells (5 × 10^5^ cells) in the 24 well plates were infected with the streptococcal strains at a multiplicity of infection (MOI) of 200, or treated with H_2_O_2_ (2 mM) for 3 h. A mixture of PMA (10 nM) and ionomycin (1 μM) (PMA + ionomycin) was used as the positive control for degranulation [34]. The supernatants were then centrifuged at 10,000 × *g* for 10 min to remove the detached cells and bacteria. The release of allergic mediators by degranulation was monitored using the β-hexosaminidase assay [35]. The clarified supernatants (50 μL) were mixed with 100 μL of substrate solution (2 mM PNAG in 0.1 M sodium citrate buffer, pH 4.5) in 96 well microtiter plates, and the mixture was incubated for 1 h at 37°C. The reaction was terminated by adding 50 μL of 2 M glycine buffer (pH 10). Absorbance at 405 nm was measured using a Multiskan FC microplate reader (Thermo Fisher Scientific, Waltham, MA, USA). The total β-hexosaminidase activity was also measured using a whole cell lysate of the RBL-2H3 cells lysed with 0.1% Triton X100. The released β-hexosaminidase activity was expressed in percentage by using the following equation:

Released hexosaminidase activity(%)=([Asamples–Ablank]/[Atotal–Ablank])×100

where *A* total is the absorbance of the reaction with the whole cell lysate, *A* sample is the absorbance of the samples, and *A* blank is the absorbance of the blank reaction mixture.

### IgE-antigen complex-triggered degranulation of RBL-2H3 cells

The RBL-2H3 cells (5 × 10^5^ cells) in 24 well plates were sensitized with mouse monoclonal anti-DNP IgE antibody (50 ng/mL) for 2 h. The cells were then washed with phosphate buffered saline (PBS; pH 7.2), cultured in a new medium containing no phenol red and antibiotics, and infected with streptococcal strains at an MOI of 10, 50 or 200, or treated with H_2_O_2_ (0.1, 0.5 or 2 mM) for 3 h. Then, the cells were stimulated for 30 min with DNP-conjugated HSA (25 ng/mL). The culture supernatants were centrifuged at 10 000 × *g* for 10 min to remove detached cells and bacteria. The hexosaminidase activity in the culture supernatants was determined as described above.

### Cell death of RBL-2H3 cells

The RBL-2H3 cells (2 × 10^5^ cells in 5% FBS DMEM) were infected with the viable streptococcal strains at an MOI of 200, in the absence of antibiotics, for 3 h. The culture medium was changed to a fresh medium containing antibiotics, and cultured for an additional 18 h. The cells were then stained with 0.2% trypan blue in PBS. After incubation at room temperature for 10 min, the numbers of viable and dead cells were counted using a microscope (Nikon TMS-F; Nikon, Tokyo, Japan). Because the dead RBL-2H3 cells were easily detached from the bottom of the culture plates, cells that disappeared during the washing and staining steps were considered to be dead (see S1 Fig). Cell death induced by H_2_O_2_ (2 mM) or PMA (10 nM) + ionomycin (1 μM) was determined similarly. To evaluate the dose-dependent effect, the cells were infected with viable *S*. *oralis* WT (MOI = 10, 50 or 200), or treated with H_2_O_2_ (0.1, 0.5 or 2 mM).

The effect of catalase was also investigated. Prior to infection, 10, 50 or 200 U/mL of catalase was added to the culture of RBL-2H3 cells, and the cells were then infected with viable *S*. *oralis* WT (MOI = 200) for 3 h. The cells were washed with PBS and cultured in fresh medium containing catalase and antibiotics for 18 h. The viability was determined as described above.

### Astra Blue staining and acidic lysosome staining

The RBL-2H3 cells were cultured on Cell Desk LF (Sumitomo Bakelite, Tokyo, Japan) in 24 well culture plates and exposed to *S*. *oralis* WT, *spxB* KO (MOI = 200), H_2_O_2_ (2 mM) or PMA + ionomycin for 3 h, changed to fresh medium containing antibiotics, and cultured for an additional 3 h (total 6 h). The cells were fixed overnight with 10% formaldehyde at 4℃ and stained with Astra Blue dye solution. The Astra Blue dye stains heparin in the granules of mast cells as blue [36].

The RBL-2H3 cells were cultured as described above, and the viable cells were stained with LysoTracker Red probe (50 nM) and SYBR Green II (1:2000 dilution) in culture medium for 15 min, washed with PBS, and observed using a Carl Zeiss Axioplan 2 fluorescent microscope system (Carl Zeiss, Oberkochen, Germany). LysoTracker Red is an acidotropic red fluorescent probe that accumulates in the acidic lysosomes. SYBR Green is a DNA-binding dyes that stains the nuclei.

### Fluorescence staining of actin

The RBL-2H3 cells were cultured on Cell Desk LF and exposed to *S*. *oralis* WT, *spxB* KO (MOI = 200), H_2_O_2_ (2 mM), or PMA + ionomycin for 3 h, washed with PBS, and cultured for an additional 3 h (total 6 h) in fresh medium containing antibiotics. The cells were fixed with 10% formaldehyde, followed by permeabilization with 0.2% Triton X-100. The DNA and actin filaments were labeled with DAPI (1 μg/mL) and AlexaFluor 594-conjugated phalloidin (1:200 dilution) in PBS for 15 min. After washing with PBS, the cell fluorescence was observed.

### Annexin V staining

The RBL-2H3 cells were cultured on Cell Desk LF and exposed to *S*. *oralis* WT, *spxB* KO (MOI = 200), H_2_O_2_ (2 mM), or PMA + ionomycin for 3 h, washed with PBS, and cultured for an additional 3 h (total 6 h) in fresh medium containing antibiotics. The cells were stained using fluoresceinisothiocyanate isomer (FITC)-Annexin V staining kit (MBL, Nagoya, Japan), according to the protocol of the manufacturer, and the fluorescence was observed using a fluorescent microscope. Differential interference contrast (DIC) images were taken in the same fields. As a positive control for apoptosis, the cells were stimulated by staurosporine (10 μM) [37] for 1 and 3 h. FITC-Annexin V (green fluorescence) binds to the apoptotic cells, and PI (red fluorescence) stains dead cells.

### Lactate dehydrogenase (LDH) assay

The RBL-2H3 cells (5 × 10^5^ cells) in 24 well plates were infected with viable *S*. *oralis* WT or *spxB* KO strains (MOI; 200) in the absence of antibiotics for 3 h. In order to stop the bacterial growth, antibiotics (penicillin [100 U/mL], and streptomycin [100 μg/mL]) were added, and the cells were cultured for additional 3 h (total 6 h). Cells were also treated with H_2_O_2_ (2 mM) or PMA + ionomycin. The supernatants were then centrifuged at 10,000 × *g* for 10 min to remove detached cells and bacteria. The LDH activity in the culture supernatants was measured using Cytotoxicity detection LDH kit (Roche Applied Science, Mannheim, Germany) according to the manufacturer’s instructions.

### Interleukin-4 (IL-4) assay

Culture supernatants of the RBL-2H3 cells exposed to viable *S*. *oralis* WT, *spxB* KO strains (MOI 200), H_2_O_2_ (2 mM) or PMA + ionomycin were assayed for IL-4 by using an enzyme-linked immunosorbent assay (ELISA) kit (Thermo Fisher Scientific) according to the manufacturer’s instructions. The cells were also sensitized with mouse anti-DNP IgE monoclonal antibody, and then with DNP-conjugated HSA, as described above.

### Effects of nasal inoculation of *S*. *oralis* and H_2_O_2_ on pollen-induced murine allergic rhinitis

The mouse experiments were performed with the approval of the animal care committee of the Osaka University Graduate School of Dentistry (No, 29-009-0). Female ddY mice (4-week-old) were purchased from Japan SLC (Hamamatsu, Japan) and fed a standard rodent diet with water *ad libitum* and maintained at 22–24°C in the animal facility at the Osaka University Graduate School of Dentistry under a 12 h/12 h light/dark cycle. To exclude the effect of the genetic background, we used the ddY inbred mice in this study. Allergic rhinitis was induced in the mice by sensitization to Japanese cedar pollen (Wako Pure Chemicals; Osaka, Japan), according to the protocols for ragweed pollen-induced allergic murine rhinitis (Fig 8A) [38, 39]. For sensitization, the mice were intraperitoneally immunized with cedar pollen (0.1 mg) with alum adjuvant (1 mg) in 200 μL of PBS on days 0, 7, and 14. The mice were further immunized by repeated intranasal administration of cedar pollen (0.5 mg/20 μL) on days 17, 18, 21–25, 31, 32, and 35–39. As a negative control, the mice were intraperitoneally administered with the alum adjuvant alone, following intranasal administration of PBS. The mice were examined for sensitization at day 42, and fully sensitized mice were subjected to further experiments. To examine the effects of *S*. *oralis* infection or H_2_O_2_ administration, the pollen-sensitized mice (4 mice per each group) were intranasally inoculated with bacterial cultures (5 × 10^7^ colony forming unit [CFU] in DMEM, 10 μL × 5 times) of *S*. *oralis* WT or *spxB* KO, or H_2_O_2_ (10 mM in DMEM, 10 μL × 5 times). The mice were challenged by intranasal inoculation of pollen (0.5 mg/20 μL) after 6 h, and the frequency of sneezing and scraping for 5 min was counted (Fig 8). The nasal inoculum (5 × 10^7^ CFU) was relevant to the murine nasal infection of *S*. *pneumoniae* [40].

### Statistical analysis

The statistical analyses were performed using QuickCalcs software (GraphPad Software, La Jolla, CA, USA) and Ekuseru Toukei (Social Survey Research Information, Tokyo, Japan). The statistical differences were examined using independent Student’s *t*-test. We also compared multiple groups using a two-tailed one-way analysis of variance (ANOVA) with Dunnett’s test. A confidence interval with a *p* value of < 0.05 was considered to be significant.

## Results

### Streptococcal infection did not induce degranulation of RBL-2H3 cells

**Fig 1:**
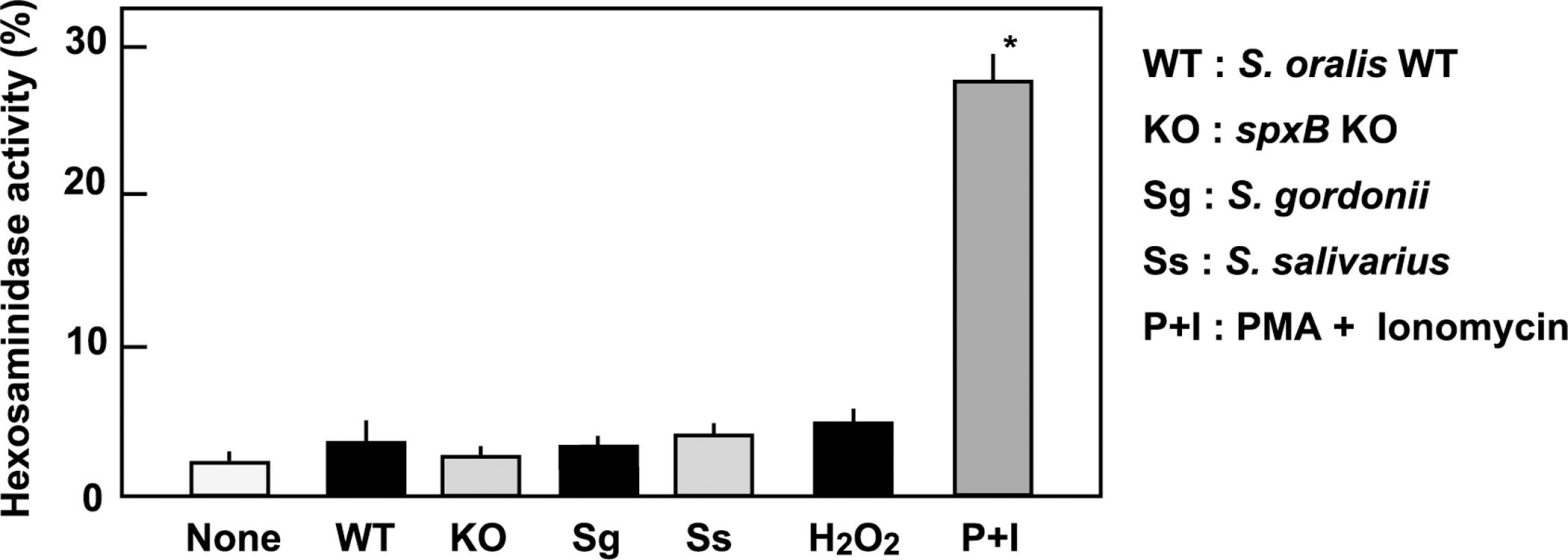
Streptococcal infection did not induce degranulation of RBL-2H3 cells. RBL-2H3 cells were infected with streptococcal strains at an MOI of 200 or treated with H_2_O_2_ (2 mM) for 3 h. A mixture of PMA (10 nM) and ionomycin (1 μM) was used as the positive control. The culture supernatants were then centrifuged to remove the detached cells and bacteria. The released β-hexosaminidase activity was determined using the PNAG substrate. The activity in the whole cell lysate was referred to as 100%. The data are shown as mean ± SD values of triplicate samples. *p < 0.05 as compared with the untreated control (None).

The rat mast cell/basophil cell line RBL-2H3 [31] was used to assess the *in vitro* effects of streptococcal infection on degranulation through a β-hexosaminidase assay [34, 35]. At first, we investigated the direct effect of streptococcal infections on RBL-2H3 cells. Viable *S*. *oralis* WT, *S*. *oralis spxB* KO (deficient for H_2_O_2_ production), *S*. *gordonii*, or *S*. *salivarius* at an MOI of 200 did not induce the release of β-hexosaminidase. Stimulation by PMA + ionomycin induced the release of β-hexosaminidase, as reported previously [34]. H_2_O_2_ (2 mM) was included in this assay, because the mitis group streptococci produce 1–3 mM of H_2_O_2_ [14, 29]. H_2_O_2_ did not elicit the release of β-hexosaminidase. These results suggest that infection with oral streptococci or exposure to H_2_O_2_ did not stimulate the degranulation of RBL-2H3 cells (Fig 1).

### Effects of streptococcal infection on IgE-induced RBL-2H3 cell degradation

**Fig 2:**
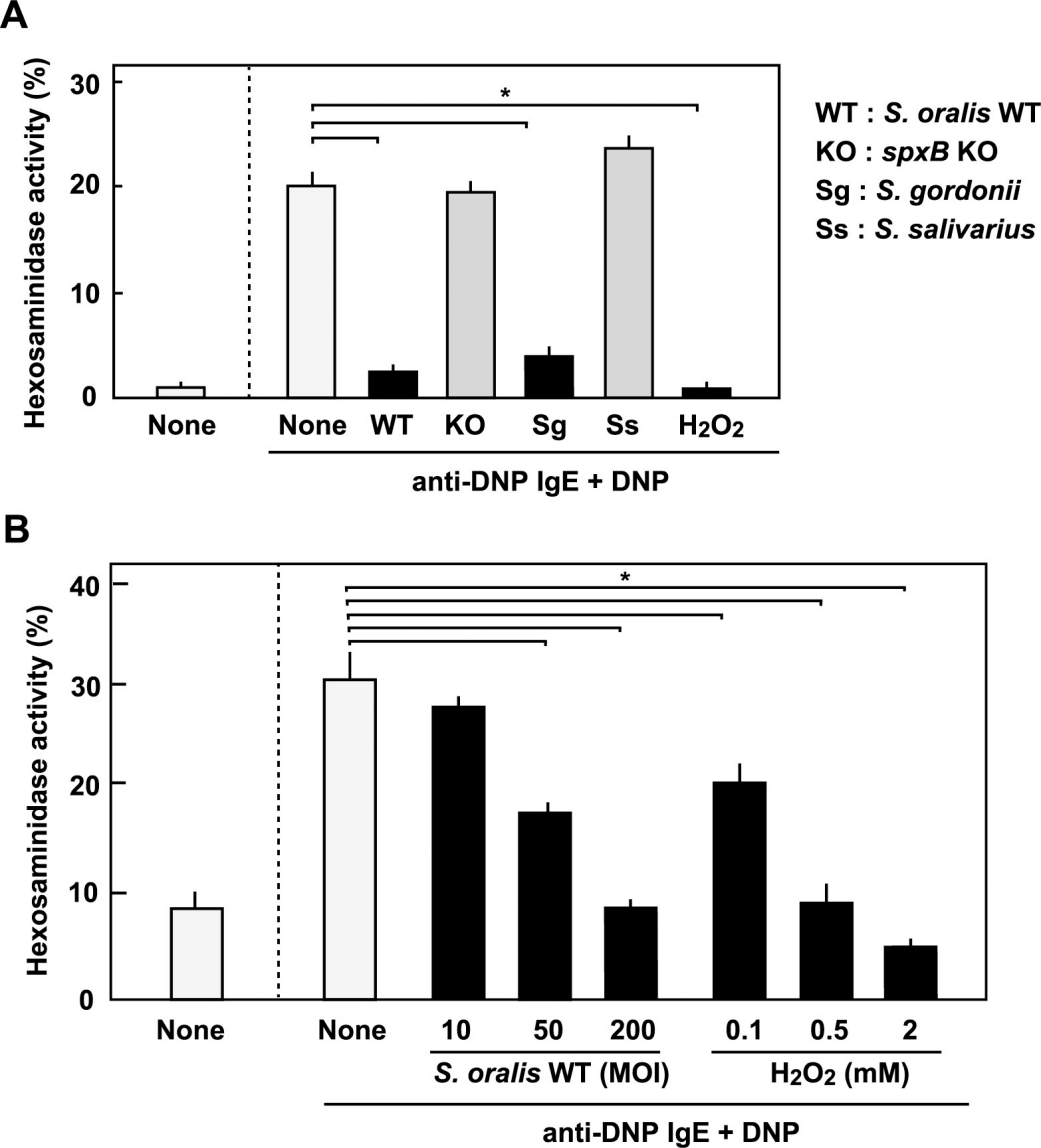
Inhibition of IgE-induced degranulation by streptococcal infection and H_2_O_2_. (**A**) RBL-2H3 cells were sensitized with mouse anti-DNP IgE monoclonal antibody, and then infected with streptococcal strains at an MOI of 200 or treated with H_2_O_2_ (2 mM) for 3 h. Then, the cells were stimulated for 30 min with DNP-conjugated HSA. The β-hexosaminidase activity in the supernatants was determined using the PNAG substrate. (**B**) RBL-2H3 cells sensitized with mouse anti-DNP IgE monoclonal antibody were infected with *S*. *oralis* WT at MOI of 10, 50, or 200 or treated with H_2_O_2_ (0.1, 0.5, or 2 mM). Then, the cells were stimulated for 30 min with DNP-conjugated HSA. The β-hexosaminidase activity in the supernatants was determined using the PNAG substrate. The activity in whole cell lysate was referred to be 100%. The data are shown as mean ± SD values of triplicate samples. *p < 0.05 as compared with the untreated IgE-stimulated control (anti-IgE + DNP, None).

Next, we investigated the effects of streptococcal infection on IgE-induced degranulation of the RBL-2H3 cells. The cells were sensitized with mouse anti-DNP IgE, and then exposed to streptococcal strains or H_2_O_2_. Then, the cells were stimulated with DNP, and the release of β-hexosaminidase was measured. Viable *S*. *oralis* WT or *S*. *gordonii* at an MOI of 200 inhibited the release of β-hexosaminidase from the RBL-2H3 cells stimulated with the IgE-antigen complex (Fig 2A). *S*. *oralis spxB* KO or *S*. *salivarius*, which does not produce H_2_O_2_, did not inhibit the IgE-triggered β-hexosaminidase release. H_2_O_2_ alone was sufficient to inhibit the degranulation (Fig 2A). These results suggest that H_2_O_2_ produced by oral mitis group streptococci inhibits IgE-stimulated degranulation.

The inhibitory effect of streptococcal H_2_O_2_ was dose-dependent (Fig 2B). The release of β-hexosaminidase decreased with increased infection of *S*. *oralis* WT, or increased concentration of H_2_O_2_ (Fig 2B).

### Streptococcal H_2_O_2_ induced the cell death of RBL-2H3 cells

**Fig 3:**
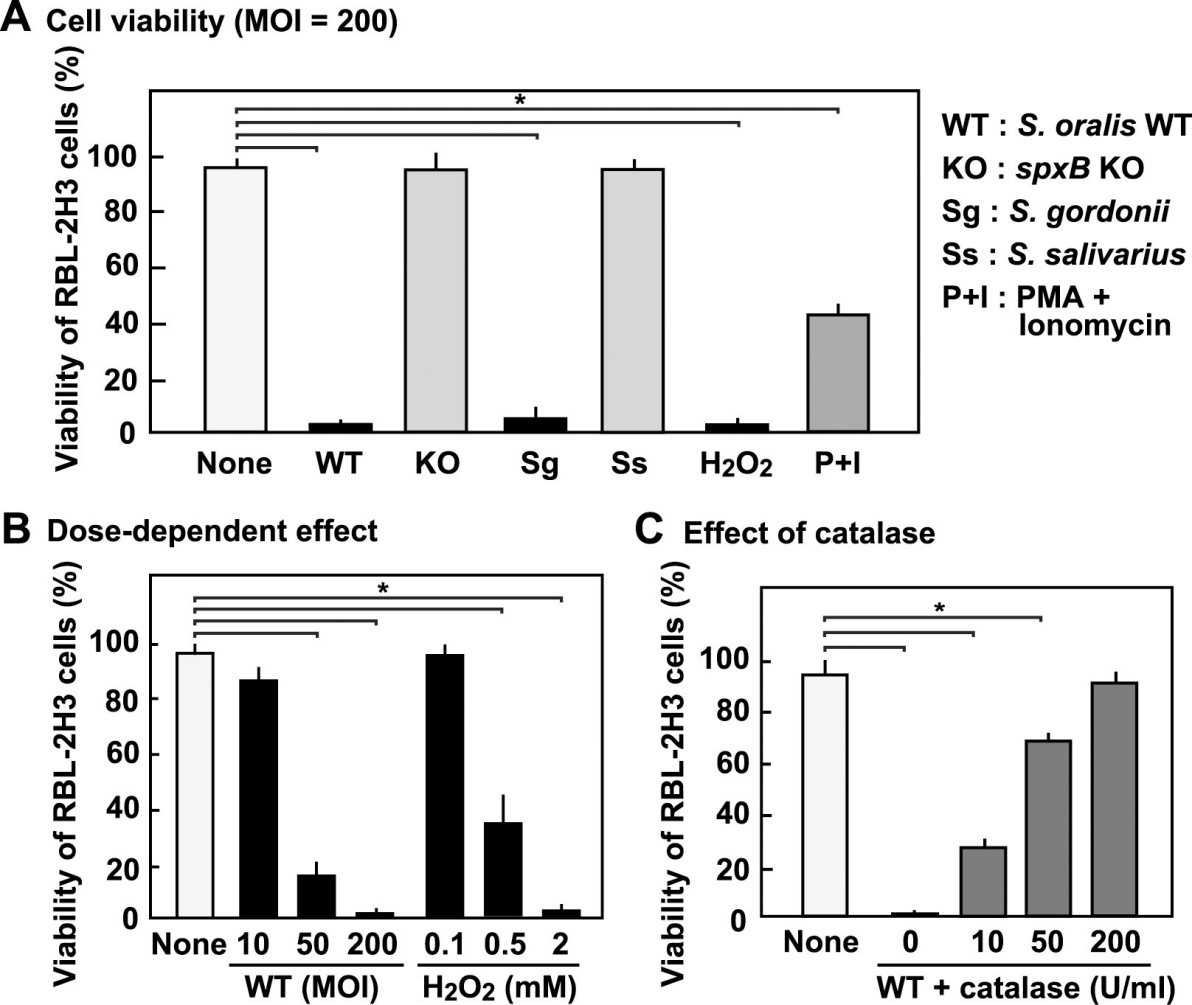
Streptococcal infection and H_2_O_2_ induce RBL-2H3 cell death. (**A**) RBL-2H3 cells were infected with viable streptococcal strains at an MOI of 200, in the absence of antibiotics, for 3 h. The cells were then cultured for 18 h in fresh medium containing antibiotics. Viability of the cells was determined using the trypan blue dye exclusion method. Viability of the cells treated with H_2_O_2_ (2 mM) or PMA (10 nM) + ionomycin (1 μM) was also determined. (**B**) To evaluate the dose dependent effect, the cells were infected with viable *S*. *oralis* WT at an MOI of 10, 50 or 200, or treated with H_2_O_2_ (0.1, 0.5 or 2 mM), and the viability was determined using the trypan blue dye exclusion method. **(C)** Involvement of streptococcal H_2_O_2_ was studied using catalase. Prior to infection, 10, 50 or 200 U/mL of catalase was added to the culture of RBL-2H3 cells, and the cells were then infected with viable *S*. *oralis* WT (MOI = 200). Viability was determined as described above. The data are shown as mean ± SD values of triplicate samples. *p < 0.05 as compared with the untreated control (None).

We had previously reported that infection with oral mitis group streptococci induces the death of macrophages, epithelial cells, and neutrophils with streptococcal H_2_O_2_ contributing to the cell death [14, 28, 29]. Therefore, we next examined whether H_2_O_2_ produced by the oral mitis group streptococci is cytotoxic to RBL-2H3 cells. The RBL-2H3 cells were exposed to viable oral streptococcal strains or H_2_O_2_, and the cells were stained with trypan blue to determine their viability (Fig 3A). Cytotoxicity of PMA + ionomycin, which induce degranulation (Fig 1), was also examined. Viable *S*. *oralis* or *S*. *gordonii* induced the cell death of the RBL-2H3 cells. Exposure to *spxB* KO or *S*. *salivarius* had little effect on the cellular viability. H_2_O_2_ was cytotoxic, and PMA + ionomycin showed moderate cytotoxicity (Fig 3A). Since infection with H_2_O_2_-producing streptococci or exposure to H_2_O_2_ resulted in the detachment of the cells from the bottom of the culture plate, we also examined the viability of the detached cells, and found that they were also dead (S1 Fig).

The dose-dependent effects of streptococcal infection and H_2_O_2_ on cellular viability were also examined. As shown in Fig 3B, streptococcal infection with MOI of more than 50 and H_2_O_2_ concentration of more than 0.5 mM were found to induce cell death of RBL-2H3 cells. Based on the dose-dependency of cytotoxicity, we additionally examined the effects of sub-cytotoxic doses of *S*. *oralis* WT (MOI = 1, 2 and 5) and H_2_O_2_ (0.01, 0.02, 0.05 mM) on the IgE-induced degranulation (S2 Fig). However, these low doses of *S*. *oralis* WT or H_2_O_2_ showed no significant effect (S2 Fig).

To examine the contribution of H_2_O_2_, we investigated the effect of catalase, an H_2_O_2_-decomposing enzyme, on *S*. *oralis*-induced cell death. Exogenously added catalase reduced death in the RBL-2H3 cells infected with *S*. *oralis* WT (Fig 3C).

### Staining for granules and lysosomes of RBL-2H3 cells

**Fig 4:**
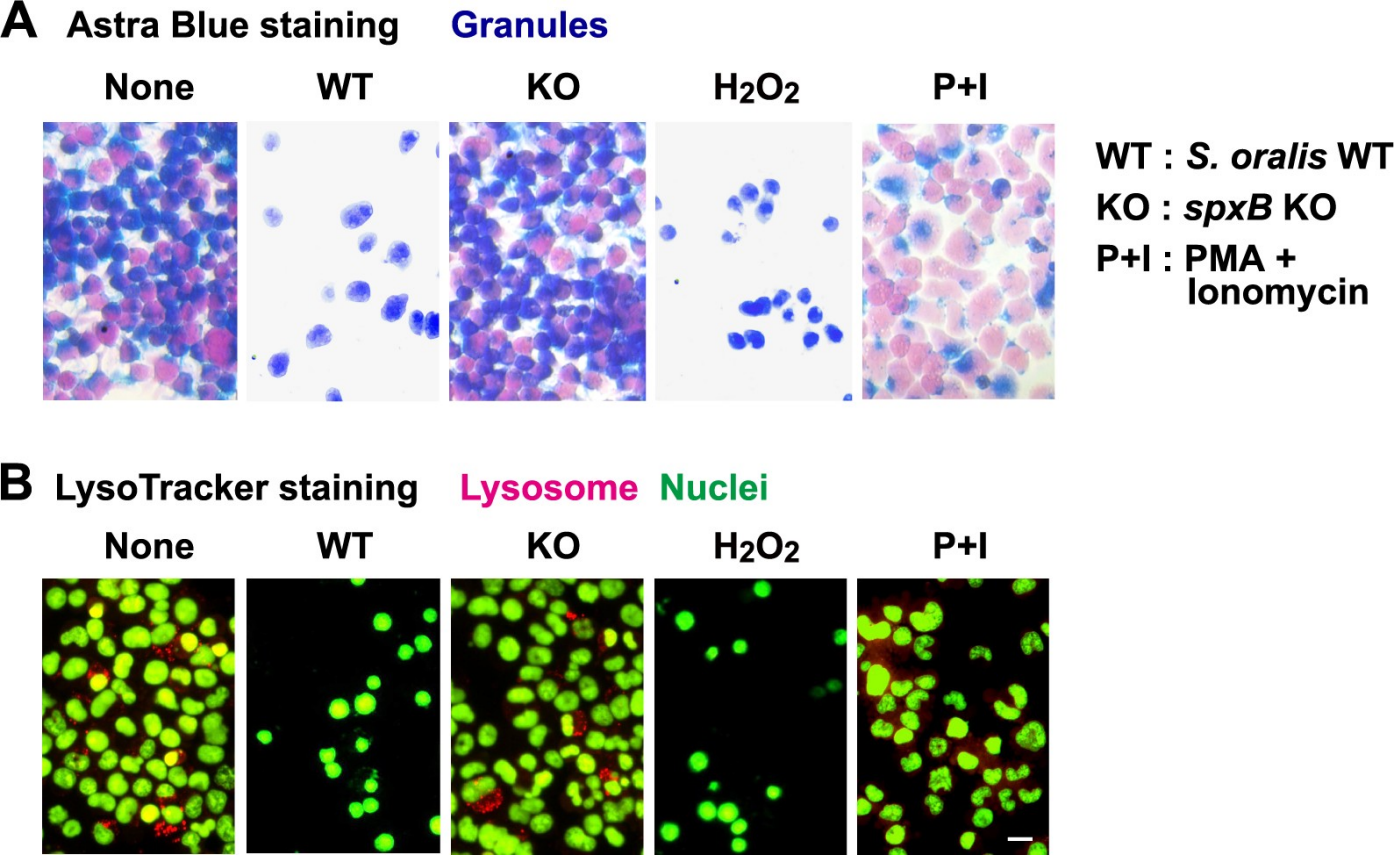
Astra Blue and acidic lysosome staining. (**A**) RBL-2H3 cells cultured on Cell Desk LF were exposed to *S*. *oralis* WT, *spxB* KO (MOI = 200), H_2_O_2_ (2 mM) or PMA (10 nM) + ionomycin (1 μM) for 3 h, and cultured for an additional 3 h (total 6 h) in fresh medium containing antibiotics. The cells were fixed with 10% formaldehyde, and stained with Astra Blue dye. (**B**) The viable cells were stained with LysoTracker Red probe and SYBR Green II, and observed using a fluorescent microscope. LysoTracker Red is a probe that accumulates to acidic lysosomes. SYBR Green stains the nuclei. Bar = 10 μm.

The granules of RBL-2H3 cells exposed to *S*. *oralis* WT, *spxB* KO or H_2_O_2_, as well as PMA + ionomycin were visualized with Astra Blue staining [36]. Many of the intact cells that contained granules were stained blue (Fig 4A, None), whereas the cells treated with PMA + ionomycin showed reduced staining because of degranulation (Fig 4A, P+I). *S*. *oralis spxB* KO did not trigger degranulation (Fig 4A, KO). The cells treated with *S*. *oralis* WT or H_2_O_2_ were stained blue **(**Fig 4A). Because the dead cells were detached from the bottom of the culture plate, the number of visible cells decreased in the cell cultures treated with *S*. *oralis* WT or H_2_O_2_.

Our previous study suggests that lysosomal damage contributes to macrophage cell death induced by H_2_O_2_ [41]. Therefore, the RBL-2H3 cells were stained with LysoTracker, an acidotropic fluorescent probe (Fig 4B). Although a limited number of LysoTracker-positive lysosomes was detectable in the RBL-2H3 cells (None), the fluorescent intensity decreased by infection with *S*. *oralis* WT or exposure to H_2_O_2_, suggesting deacidification of the lysosomes occurred during the bacterial infection or exposure to H_2_O_2_. In addition, lysosomal damage was not observed in *S*. *oralis spxB* KO-infected cells.

### Fluorescence staining of actin of RBL-2H3 cells

**Fig 5:**
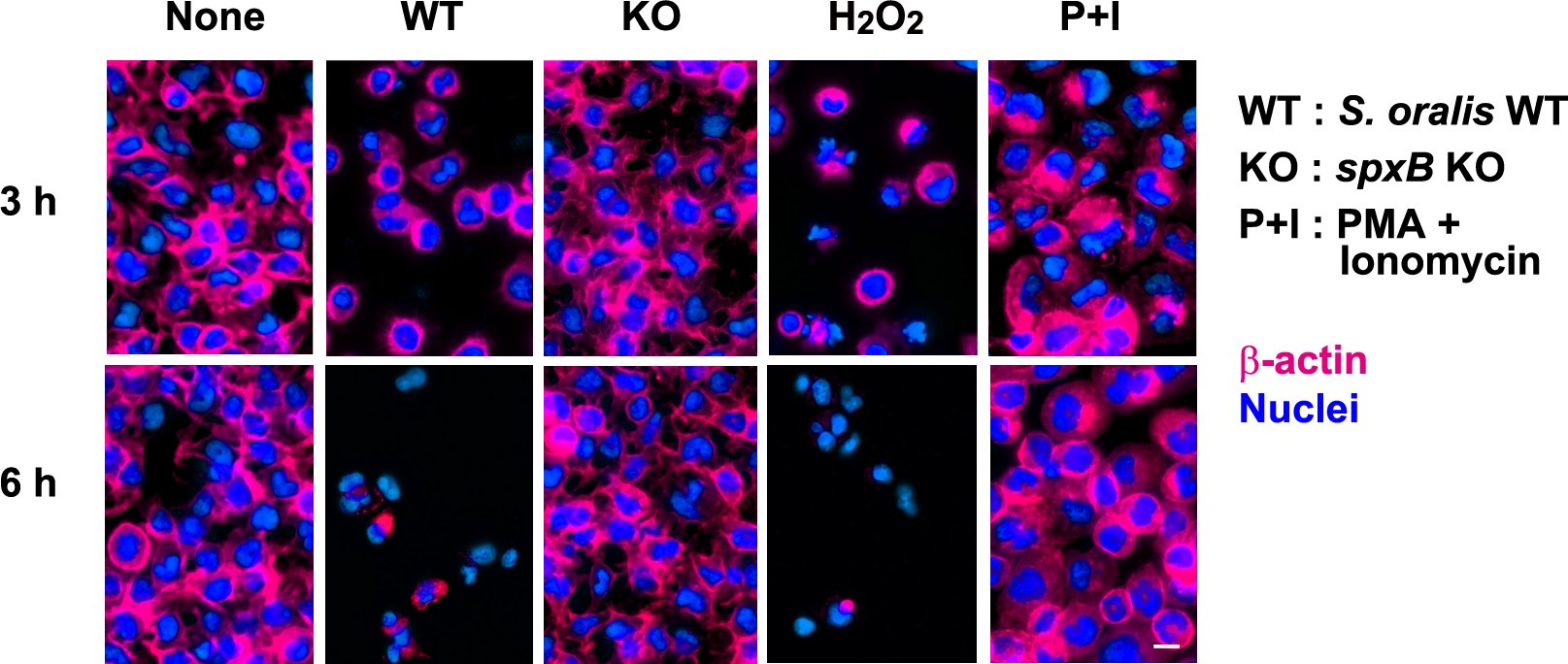
Fluorescence staining of actin. RBL-2H3 cells on the Cell Desk were exposed to *S*. *oralis* WT, *spxB* KO (MOI = 200), H_2_O_2_ (2 mM) or PMA (10 nM) + ionomycin (1 μM) for 3 h (upper images), and cultured for an additional 3 h (total 6 h; lower images) in fresh medium containing antibiotics. The cells were fixed with 10% formaldehyde, followed by permeabilization with 0.2% Triton X-100. The DNA and actin filaments were labeled with DAPI and AlexaFluor 594-conjugated phalloidin. Bar = 10 μm.

We found that the dead RBL-2H3 cells easily detached from the bottom of the culture plate. Therefore, we examined the actin filaments in the RBL-2H3 cells treated with *S*. *oralis* WT, *spxB* KO or H_2_O_2_ (Fig 5). Immunofluorescence staining of actin showed decreased actin filaments in the RBL-2H3 cells exposed to *S*. *oralis* WT or H_2_O_2,_ whereas *S*. *oralis spxB* KO did not induce such morphological change (Fig 5).

### Annexin V staining and release of LDH

**Fig 6:**
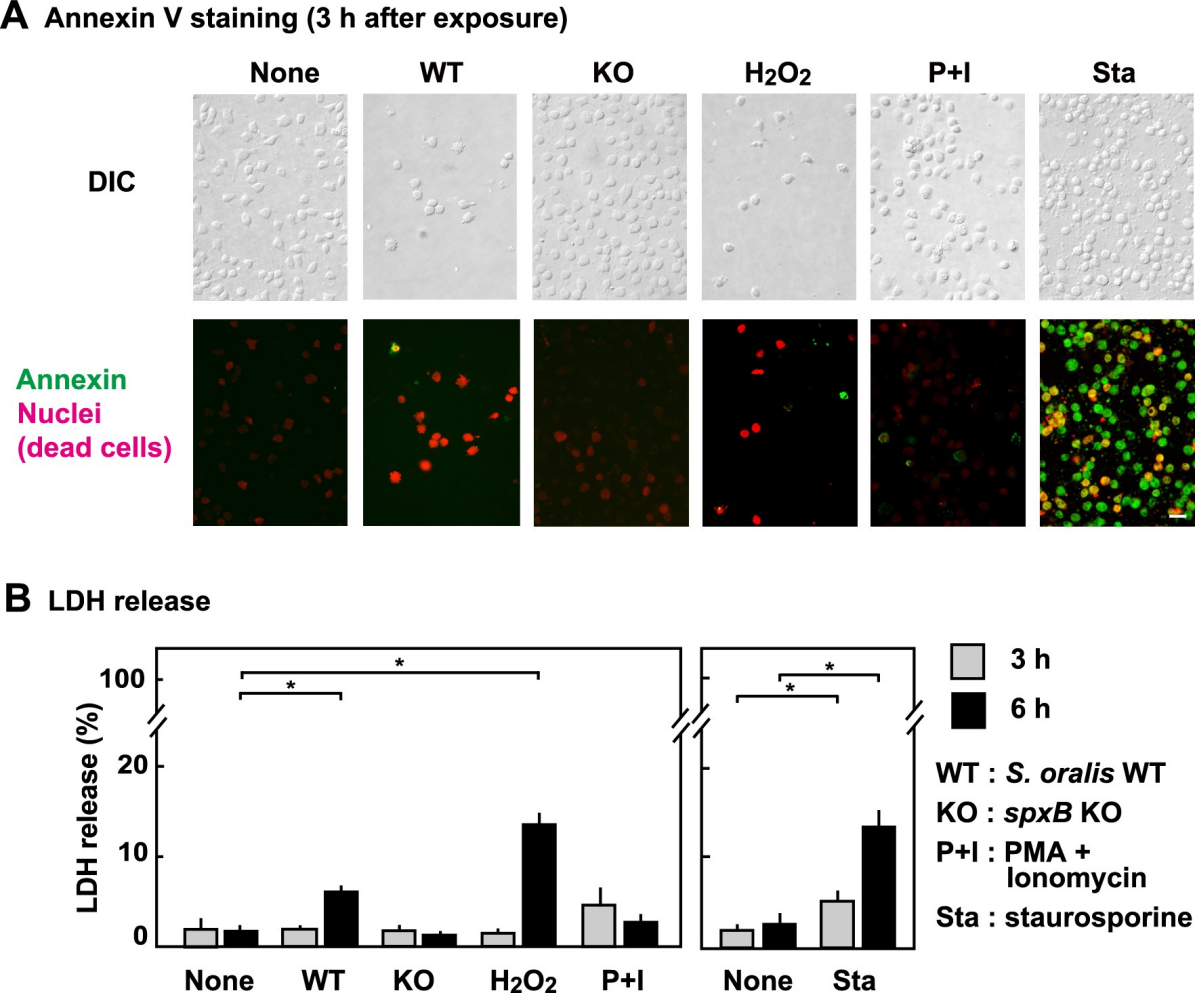
Annexin V fluorescent staining and LDH release. (**A**) The RBL-2H3 cells were cultured on Cell Desk LF and exposed to *S*. *oralis* WT (MOI = 200), H_2_O_2_ (2 mM), or PMA (10 nM) + ionomycin (1 μM) for 3 h, and were stained using FITC-Annexin V staining kit. Staurosporine (10 μM) was used as a positive control for apoptosis. FITC-Annexin V (green fluorescence) binds to the apoptotic cells, and PI (red fluorescence) stains dead cells (lower images). Differential interference contrast (DIC) images were taken in the same fields (upper images). Bar = 20 μm. (**B**) RBL-2H3 cells (5 × 10^5^ cells) in 24 well plates were infected with viable *S*. *oralis* WT (MOI = 200) in the absence of antibiotics for 3 h. Then, the infected cells were cultured in the presence of antibiotics for an additional 3 h (total 6 h). The cells were also treated with H_2_O_2_ (2 mM), PMA (10 nM) + ionomycin (1 μM) or staurosporine (10 μM). The culture supernatants were then centrifuged to remove the detached cells and bacteria. LDH in the culture supernatants was measured using an LDH activity assay kit according to the manufacturer’s instructions.

To evaluate whether streptococcal infection or H_2_O_2_ exposure induces necrotic or apoptotic cell death, FITC-Annexin V staining and LDH release were investigated (Fig 6). Annexin V is known to bind to apoptotic cells, and thus, the apoptotic cells show green fluorescence. PI (red fluorescence) stains all dead cells. Cells treated with staurosporine, an apoptosis inducer [37], were stained by both Annexin V and PI. However, Annexin V did not bind to the RBL-2H3 cells exposed to *S*. *oralis* WT or H_2_O_2_ (Fig 6A). These cells were stained by PI, indicating that they were dead. Infection by *S*. *oralis spxB* KO or exposure to PMA + ionomycin did not induce cell death (Fig 6A). These results suggested that the cell death induced by strepotococcal H_2_O_2_ was not apoptosis.

LDH release from dead cells is used as an indicator of necrotic cell death. Cells undergoing apoptotic death do not release LDH in their early cell death stage, because the cell membranes are intact. At the early stage (3 h), infection by *S*. *oralis* WT, or exposure to H_2_O_2_ did not stimulate LDH release from the RBL-2H3 cells. However, increased LDH release at 6 h after exposure to *S*. *oralis* WT or H_2_O_2_ was observed (Fig 6B, left). Cells treated with staurosporine gradually released LDH in the culture medium (Fig 6B, right).

### IL-4 release from RBL-2H3 cells

**Fig 7:**
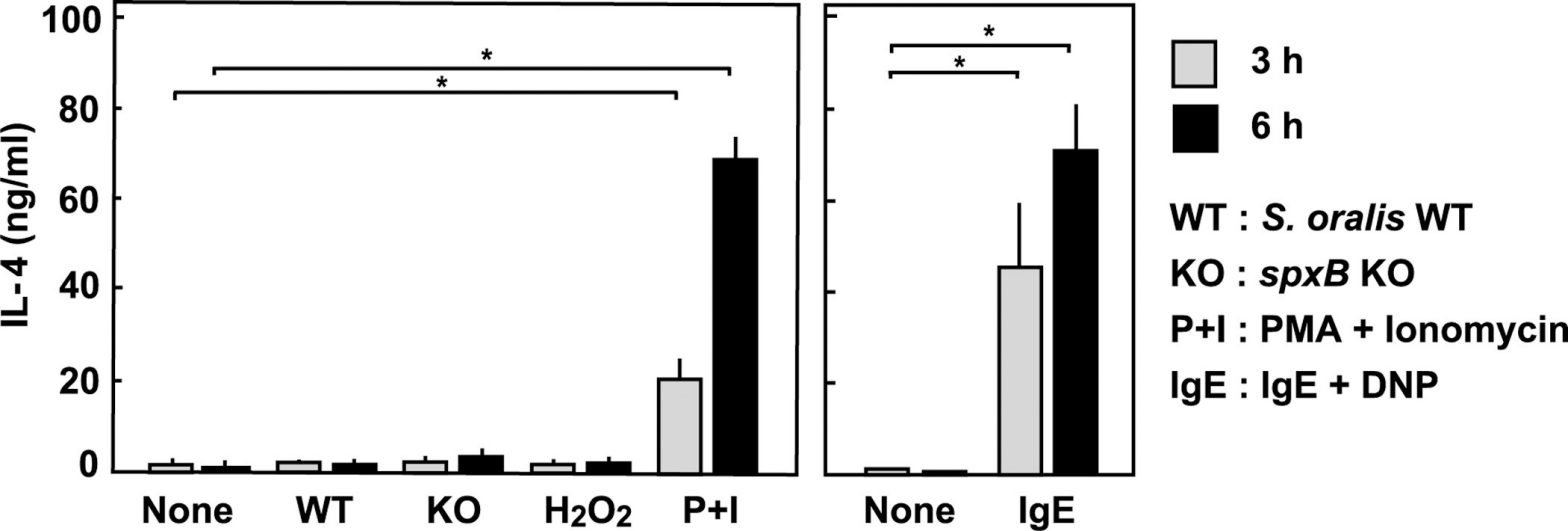
IL-4 production from RBL-2H3 cells. RBL-2H3 cells (5 × 10^5^ cells) in 24 well plates were infected with viable *S*. *oralis* WT (MOI = 200) in the absence of antibiotics for 3 h. Then, the infected cells were cultured in the presence of antibiotics for an additional 3 h (total 6 h). The cells were also treated with H_2_O_2_ (2 mM) or PMA (10 nM) + ionomycin (1 μM). The cells were also sensitized with mouse anti-DNP IgE monoclonal antibody, and then with DNP-conjugated HSA. The culture supernatants were then centrifuged to remove the detached cells and bacteria. The amount of IL-4 in the culture supernatants was measured using an ELISA kit. The data are shown as mean ± SD values of triplicate samples. *p < 0.05 as compared with the untreated control (None).

Activated mast cells and basophils are reported to produce IL-4 [15, 16, 18, 21]. Thus, IL-4 in the culture medium of the treated cells was also measured (Fig 7). Infection by *S*. *oralis* WT or *spxB* KO, or exposure to H_2_O_2_ did not stimulate IL-4 production from the RBL-2H3 cells, whereas PMA + ionomycin induced its production (Fig 7, left). As well as PMA + ionomycin, activation by IgE-antigen complex stimulated IL-4 production (Fig 7, right). We also found that sub-cytotoxic doses of *S*. *oralis* WT or H_2_O_2_ showed no significant effect on Il-4 production from RBL-2H3 cells (S3 Fig).

### *In vivo* study of the effects of nasal inoculation of *S*. *oralis* and H_2_O_2_ on pollen-induced murine allergic rhinitis

**Fig 8:**
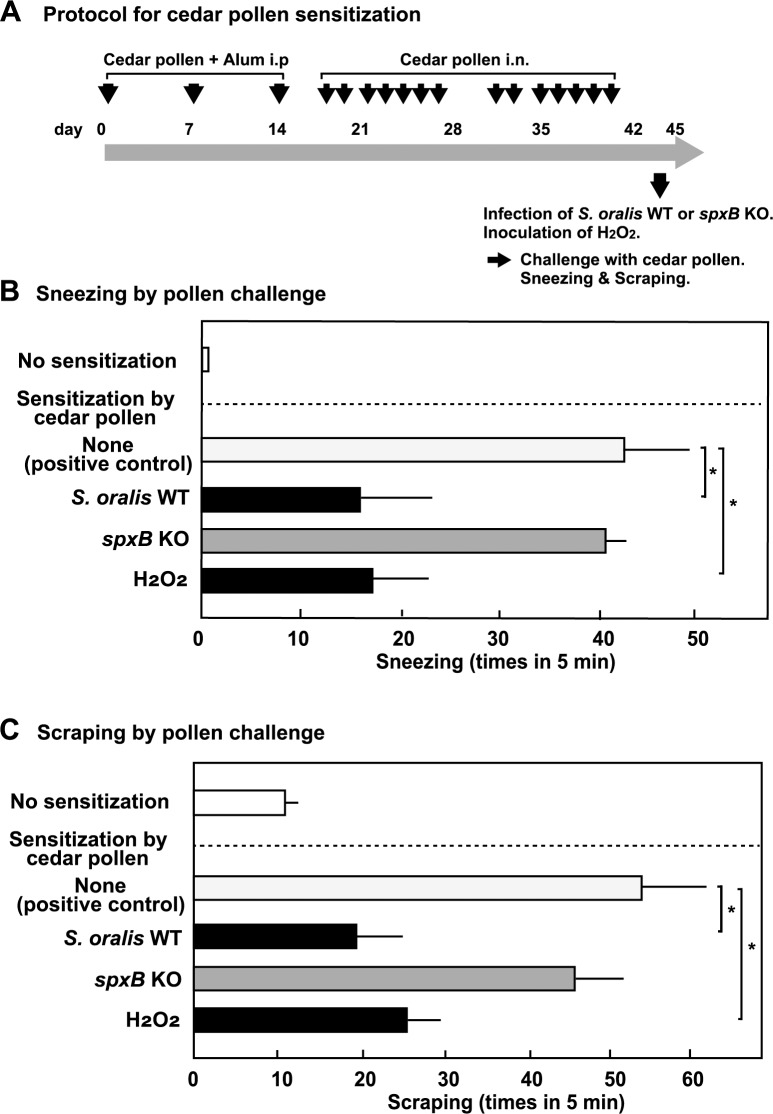
Effect of nasal inoculation of *S*. *oralis* and H_2_O_2_ on pollen-induced murine allergic rhinitis. (**A**) The mice were intraperitoneally immunized with cedar pollen (0.1 mg) with alum adjuvant (1 mg) in 200 μL of PBS three times, and further immunized by repeated intranasal administration of cedar pollen (0.5 mg/20 μL). The mice were examined for sensitization on day 42, and the fully sensitized mice were subjected to further experiments. The pollen-sensitized mice (4 mice per each group) were intranasally inoculated with bacterial cultures (5 × 10^7^ CFU in DMEM, 10 μL × 5 times) of *S*. *oralis* WT, *spxB* KO, or H_2_O_2_ (10 mM in DMEM, 10 μL × 5 times). The mice were challenged by intranasal inoculation of pollen (0.5 mg/20 μL) after 6 h, and the frequency of sneezing (**B**) and scraping (**C**) for 5 min was counted. The data are shown as mean ± SD values of 4 mice. *p < 0.05 as compared with the positive control (None, pollen challenge alone).

To confirm the effect of streptococcal H_2_O_2_ on *in vivo* allergic reaction, a murine pollen-induced allergic rhinitis model [38, 39] was used. Repeated immunization of mice with cedar pollen successfully induced allergic rhinitis (Fig 8). Then, the sensitized mice were intranasally inoculated with *S*. *oralis* culture or H_2_O_2_. As compared with positive control mice and mice infected with *spxB* KO strain, mice either infected by *S*. *oralis* WT or exposed to H_2_O_2_ exhibited the reduced number of sneezes and scraping after the pollen challenge, suggesting that the inhibition of the allergic reaction is mediated by H_2_O_2_ (Fig 8). However, underlying mechanisms remain to be conclusively defined.

## Discussion

**Fig 9:**
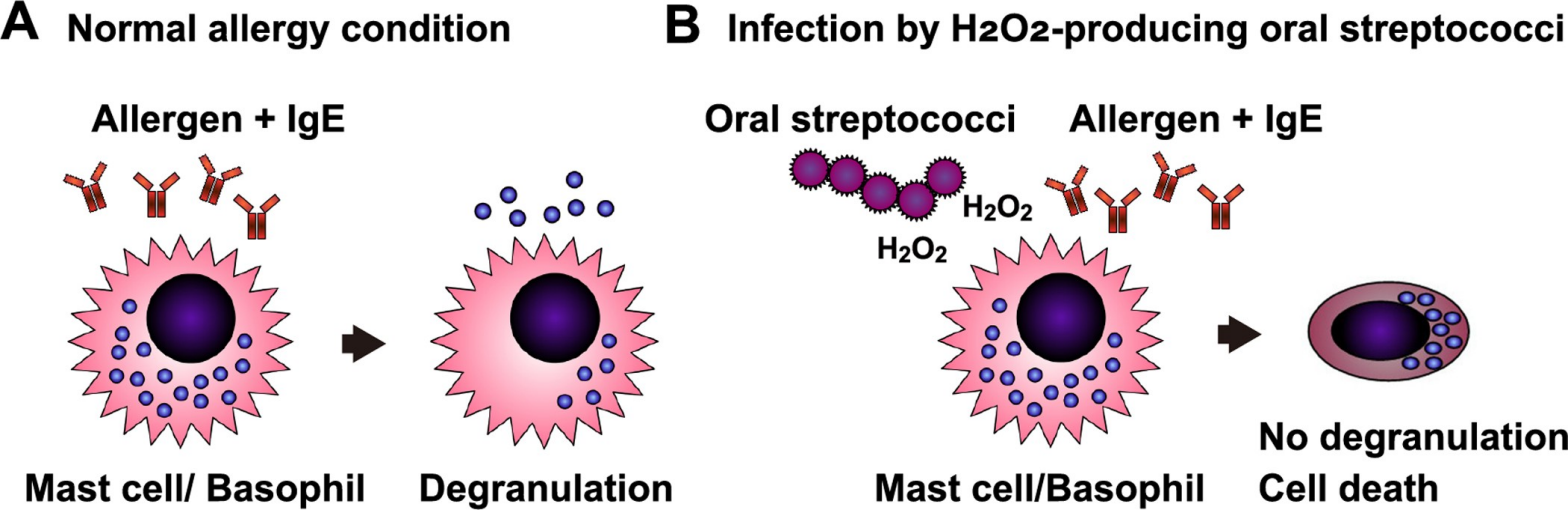
Summary of this study. (**A**) Under normal allergy conditions, allergen-binding IgE induces degranulation from mast cells. (**B**) Infection by H_2_O_2_-producing oral mitis group streptococci causes the death of mast cells. The IgE-antigen complex cannot induce the degranulation of the streptococci-infected mast cells.

This study reveals that H_2_O_2_ produced by oral mitis group streptococci inhibits degranulation of the RBL-2H3 mast cells/basophils stimulated by the IgE-allergen complex. The cytotoxicity of the streptococcal H_2_O_2_ contributes to the inhibition of degranulation. The results are summarized in Fig 9.

In our previous studies, we have shown that H_2_O_2_ produced by oral mitis group streptococci is cytotoxic to host innate immune cells such as macrophages, neutrophils, and epithelial cells [14, 27, 28, 29]. Moreover, we recently found that streptococcal H_2_O_2_ induces a stress response in macrophages [30]. Such stress responses could contribute to the suppression of degranulation in mast cells/basophils. Other studies have also reported the cytotoxicity and pathogenicity of streptococcus-derived H_2_O_2_ [42, 43, 44, 45, 46]. These findings suggest that streptococcal H_2_O_2_ has beneficial effects on streptococcal colonization of the mucosal surfaces and even on infections leading to cardiovascular diseases.

Infection with *S*. *pneumoniae* is reported to activate mast cells [23, 24]. Fritscher et al. [24] showed that the activation of mast cells is dependent on a pore-forming cytolysin named pneumolysin [47]. Other studies have shown that pyrogenic exotoxin of *S*. *pyogenes* and hemolytic lipid toxin of *S*. *agalactiae* stimulate the degranulation of mast cells [25, 26]. These studies also suggest that pathogenic streptococci modulate mast cell function to evade the host immune response.

Regarding to the effect of H_2_O_2_ on the mast cells, Peden et al. [48] reported that H_2_O_2_ (0.2–2 mM) inhibits cell proliferation and IgE-induced degranulation of the RBL-2H3 cells. The concentration of H_2_O_2_ in their study was similar to the concentration in the culture supernatants of *S*. *oralis* WT (1–3 mM) [14, 29]. Other studies also have reported that exogenous H_2_O_2_ impairs the degranulation of mast cells [49, 50]. In contrast, intracellular reactive oxygen species produced by mitochondria have been reported to contribute to the degranulation and cytokine production of mast cells [51]. These studies do not address the cytotoxicity of H_2_O_2_ against mast cells, whereas our present study clearly demonstrated that the cytotoxicity of streptococcal H_2_O_2_ plays an important role in the inhibition of the IgE-induced degranulation.

To test the potential effects of streptococcal H_2_O_2_ on *in vivo* allergic reaction, we used a murine pollen-induced allergic rhinitis model (Fig 8). Pretreatment with streptococcal culture or H_2_O_2_ resulted in a significant reduction of the allergic reaction, such as sneezing and scraping, after the pollen challenge (Fig 8). Thus, streptococcal H_2_O_2_ may reduce the allergic reaction through its suppressive effect on mast cells and basophils. Possibly, suppression of other immune cells such as macrophages [41] and neutrophils [29] is involved in the reduction of the allergic reaction. It should be noted that due to non-negligible variation in methods of *in vivo* pollen-induced rhinitis model conducted by each group [38, 39], it is difficult to compare our results to those of previous studies. For example, Haenuki et al. [38] sensitized mice of C57BL/6 background with ragweed pollen, while Kato et al. [39] sensitized BALB/c mice. To exclude the effect of the genetic background, we used ddY inbred mice in this study, and the level of the allergic response such as sneezing is similar to those of their results. Although *S*. *oralis* is not considered to be major inhabitant of the nasal cavity, a study using human specimen shows that the *Streptococcus* genus was represented in nasal cavity and *S*. *oralis/Streptococcus mitis* (these two species are very similar) was most prevalent members (17.6%) [52]. *S*. *pneumoniae* is one of the residents of the nasal cavity [53, 54], and therefore, pneumococcal H_2_O_2_ is likely to damage the immune response in the nasal cavity [42, 43, 44]. To appraise the contribution of nasal and/or oral mitis group streptococci to the inhibition of the allergic response, more detailed analysis including human clinical studies will be required.

Recent studies have revealed that mast cells and basophils control the innate immune response in various ways. IgE-induced immune response and degranulation from mast cells and basophils are considered to be host defense responses against parasites [15, 16, 18, 19, 21, 55]. In this study, we found that H_2_O_2_ produced by mitis group streptococci inhibits the IgE-induced degranulation from RBL-2H3 cells. Moreover, streptococcal H_2_O_2_ does not stimulate the production of IL-4 from the cells. Such suppressive effect of the innate immune response is considered to be an evasion strategy of streptococci to escape from the host immune response, thereby supporting streptococcal colonization at the mucosal surface of the oral cavity [30, 55]. In this regard, previous studies have suggested that inhibition of degranulation of mast cells prevents both inflammatory and allergic responses [56, 57]. Interestingly, commensal bacteria are necessary for the maintenance of a healthy mucosal immune system. Commensal *Clostridia* can contribute to immune homeostasis in the intestine by inducing the differentiation of regulatory T cells through synthesis of short-chain fatty acids such as butyrate [58]. On the other hand, a recent study suggested that microbial oxygen respiration contributes to intestinal inflammation [59]. H_2_O_2_ from oral streptococcus may have such potential immunomodulatory effect on the innate immune system in the oral cavity.

We had previously reported that streptococcal H_2_O_2_ induces the death of macrophages, and dysfunction of lysosomes contributes to cell death [41]. Lysosomes are organelles filled with “cytotoxic” hydrolytic enzymes, including proteases, and their dysfunction is considered to induce cell death [60, 61]. Therefore, we examined the effect of streptococcal H_2_O_2_ on lysosomal integrity of RBL-2H3 cells (Fig 4). LysoTracker fluorescent staining demonstrated that the streptococcal H_2_O_2_ elicited a reduction in the acidic lysosomal environment. Effect of deferoxamine, which is an iron chelator and reduces the production of peroxide radicals from H_2_O_2_ within lysosomes [62, 63], also suggests the involvement of lysosomal dysfunction in the cell death of mast cells (S4 Fig).

Braun et al. [43] and Rai et al. [45] have revealed that pneumococcal H_2_O_2_ induces apoptosis in microglia and lung cells, respectively. However, in our study, the involvement of apoptotic event in H_2_O_2_-induced RBL-2H3 cell death was not evident (Fig 6). The dead RBL-2H3 cells were Annexin V-negative, suggesting that their cell death is not apoptosis. It should be noted that the H_2_O_2_-induced cell death was not accompanied with degranulation, thereby inducing limited inflammatory response. In this study, we used only one cell line, RBL-2H3, and therefore our findings have to be confirmed using other cell lines or primary mast cells in future studies.

In summary, our results reveal that H_2_O_2_ produced by oral mitis group streptococci inhibit the degranulation of mast cells/basophils through the induction of cell death, suggesting that H_2_O_2_ plays a significant role in the modulation of the innate immune response induced by mast cells in the mucosal surface. Effects of streptococcal H_2_O_2_ on the local allergic response such as pollen-induced rhinitis or metal allergy will be of special interest for future studies.

## Supporting information

S1 Fig Trypan blue staining of detached RBL-2H3 cells. (TIFF)

S2 Fig Effects of sub-cytotoxic doses of *S*. *oralis* and H_2_O_2_ on IgE-induced degranulation of RBL-2H3 cells. (TIFF)

S3 Fig Effects of sub-cytotoxic doses of S. oralis and H_2_O_2_ on IL-4 production of RBL-2H3 cells. (TIFF)

S4 Fig Effect of deferoxamine (DFO) on the cell death of RBL-2H3 cells. (TIFF)

S1 Appendix Supplementary Materials and Methods, Results and Discussion for S4 Fig. (PDF)

S2 Appendix Minimal data set. Values used to build graphs.
(PDF)
