# Supplementary figures and images for: Streptococcal H2O2 inhibits IgE-triggered degranulation of RBL-2H3 mast cell/basophil cell line by inducing cell death
Source: PLoS One. 2020 Apr 17;15(4):e0231101. doi: 10.1371/journal.pone.0231101 (PMC7164662; doi:10.1371/journal.pone.0231101)

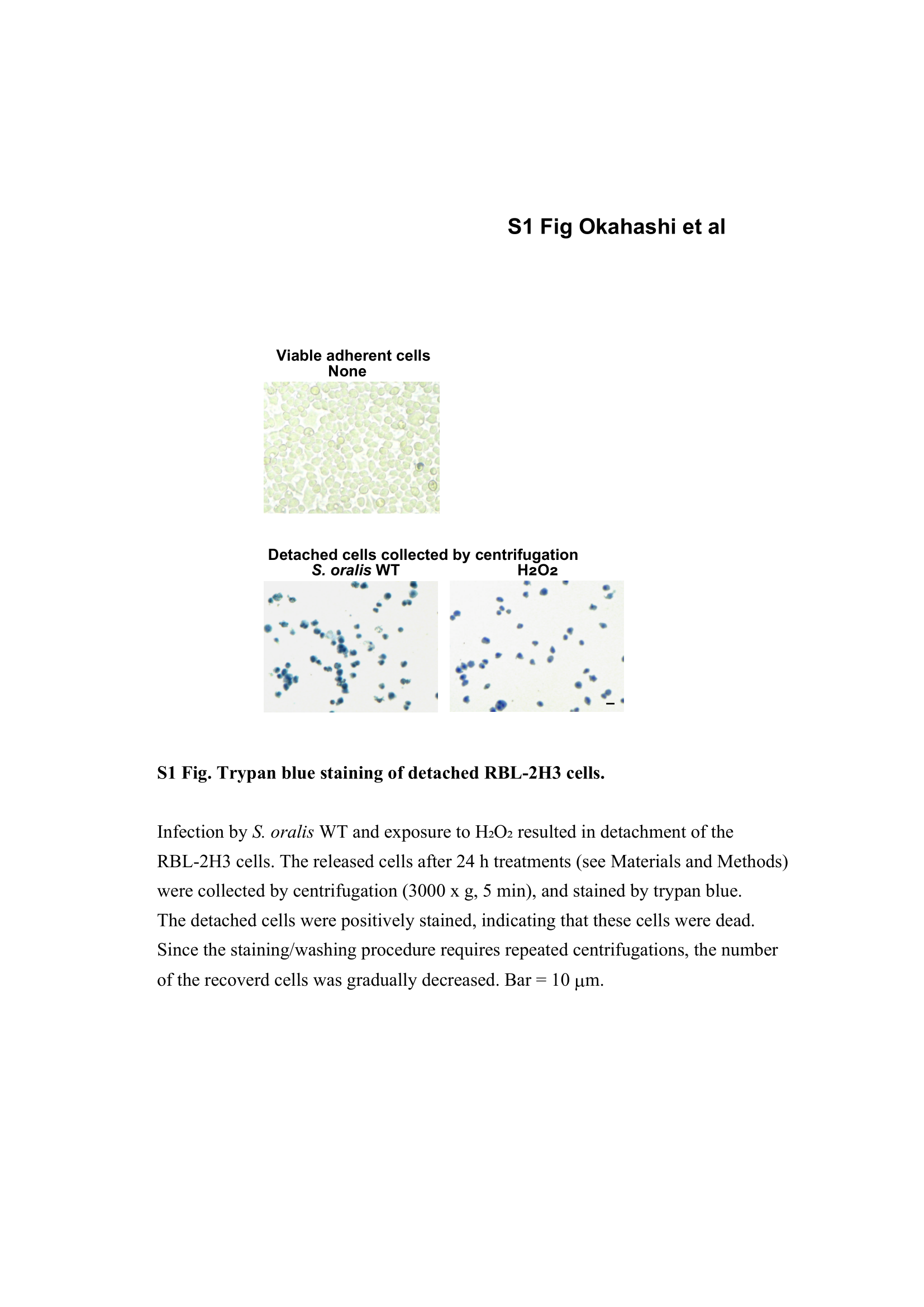

Supplement: S1 Fig — (TIFF) [file pone.0231101.s001.tiff]

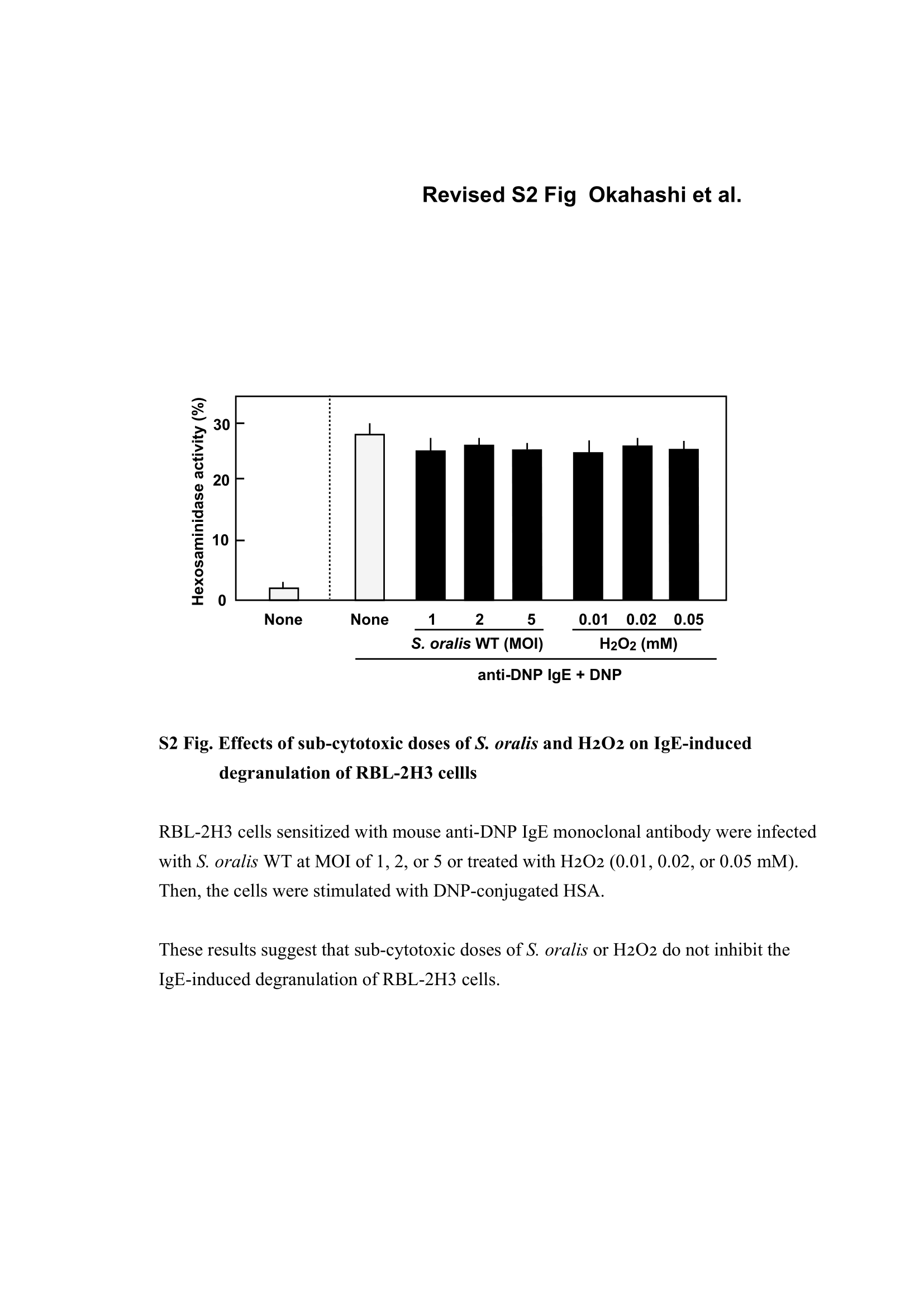

Supplement: S2 Fig — (TIFF) [file pone.0231101.s002.tiff]

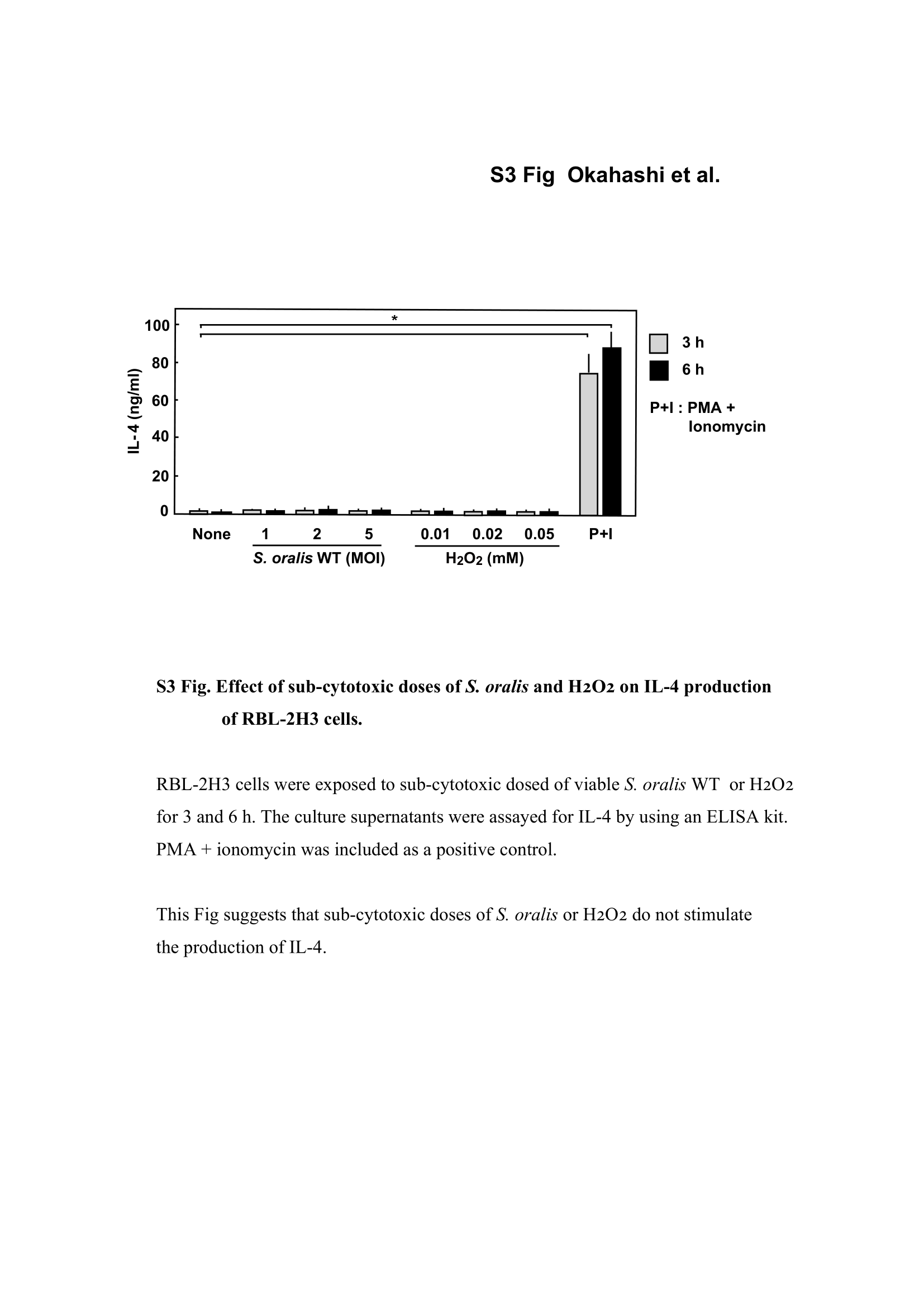

Supplement: S3 Fig — (TIFF) [file pone.0231101.s003.tiff]
