# Supplementary material for: Streptococcal H2O2 inhibits IgE-triggered degranulation of RBL-2H3 mast cell/basophil cell line by inducing cell death
Source: PLoS One. 2020 Apr 17;15(4):e0231101. doi: 10.1371/journal.pone.0231101 (PMC7164662; doi:10.1371/journal.pone.0231101)
Supplement: S1 Appendix — (PDF) [file pone.0231101.s005.pdf]

S1 Appendix Materials and Methods, Results and Discussion for S4 Fig.

Matrials and Methods for S4 Fig.

Prior to bacterial infection or H_2O_2 treatment, the RBL-2H3 cells were treated with 0.5, 2, or 5 mM of deferoxamine (DFO) mesylate (a bacterial siderophore that chelates iron [Fe]; Sigma-Aldrich). The cells were then infected with viable *S. oralis* strains (MOI = 200) or treated with 2 mM H_2O_2 for 3 h. Cells were washed with PBS, and cultured in fresh medium containing antibiotics for 21 h. The viability was determined using the trypan blue staining.

Results and Discussion for S4 Fig.

DFO is an iron [Fe] chelator, and it has been also reported to reduce the production of peroxide radicals from H_2O_2 within lysosomes [62, 63]. This Fig. shows that DFO reduced the cytotoxicity of streptococcal H_2O_2, suggesting that oxygen radicals from H_2O_2 contribute to the lysosomal dysfunction and death of mast cells.
