# Supplementary material for: Streptococcal H2O2 inhibits IgE-triggered degranulation of RBL-2H3 mast cell/basophil cell line by inducing cell death
Source: PLoS One. 2020 Apr 17;15(4):e0231101. doi: 10.1371/journal.pone.0231101 (PMC7164662; doi:10.1371/journal.pone.0231101)
Supplement: S2 Appendix — Values used to build graphs. (PDF) [file pone.0231101.s006.pdf]

S2 Appendix Minimal data set. Values used to build graphs

Values for Fig 1

Sample	Hexosaminidase activity OD405		
Blank	0.082	0.083	0.082
Whole cell lysate	1.271	1.42	1.223
After stimulation			
None	0.135	0.138	0.159
WT	0.154	0.168	0.243
KO	0.13	0.144	0.16
Sg	0.159	0.15	0.157
Ss	0.157	0.163	0.191
H2O2	0.178	0.191	0.198
P+I	0.762	0.686	0.77

Values for Fig 2A

Sample	Hexosaminidase activity OD405		
Blank	0.049	0.048	0.05
Whole cell lysate	1.611	1.786	1.637
None	0.076	0.076	0.09
After IgE-stimulation			
None	0.399	0.371	0.366
WT	0.136	0.167	0.084
KO	0.275	0.391	0.386
Sg	0.101	0.115	0.156
Ss	0.416	0.416	0.442
H2O2	0.061	0.075	0.079

Values for Fig 2B

Sample	Hexosaminidase activity OD405		
Blank	0.055	0.063	0.059
Whole cell lysate	0.492	0.449	0.529
None	0.081	0.105	0.104
After IgE-stimulation			

None		0.178	0.206	0.185
WT	10	0.182	0.188	0.169
	50	0.137	0.139	0.129
	200	0.098	0.101	0.096
H2O2	0.1	0.133	0.138	0.161
	0.5	0.085	0.095	0.116
	2	0.063	0.089	0.09

Values for Fig 3A

Sample	Number of viable cell/microscopic area		
None	118	121	114
WT	0	0	0
KO	105	109	129
Sg	11	18	5
Ss	121	113	109
H2O2	0	0	1
P+I	57	40	51

Values for Fig 3B

Sample		Number of viable cell/microscopic area		
None		118	139	122
WT	10	106	98	107
	50	14	30	23
	100	0	0	0
H2O2	0.1	129	125	118
	0.5	59	35	45
	2	0	0	0

Values for Fig 3C

Sample		Number of viable cell/microscopic area		
None		102	115	110
WT		0	3	3
	catalase 10 U	27	30	34

catalase 50 U	71	83	74
catalase 100 U	97	99	105

Values for Fig 6B

Sample	LDH release OD492		
Blank	0.051	0.05	0.053
Whole cell lysate	1.975	2.206	2.043
None 3 h	0.191	0.179	0.18
None 6 h	0.18	0.184	0.161
WT 3 h	0.213	0.194	0.176
WT 6 h	0.264	0.265	0.275
KO 3 h	0.187	0.175	0.171
KO 6 h	0.144	0.148	0.155
H2O2 3 h	0.181	0.175	0.15
H2O2 6 h	0.416	0.401	0.417
P+I 3 h	0.285	0.286	0.332
P+I 6 h	0.257	0.282	0.187

Blank	0.072	0.06	0.059
Whole cell lysate	0.578	0.641	0.673
None 3 h	0.089	0.061	0.081
None 6 h	0.083	0.092	0.083
Stau 3 h	0.101	0.119	0.107
Stau 6 h	0.124	0.163	0.13

Values for Fig 7

Sample	IL-4 ng/ml		
None 3 h	0.5	0	1.9
None 6 h	0.2	0.4	1.3
WT 3 h	1	2.2	1.7
WT 6 h	0.3	0.7	0.2
KO 3 h	2.8	1.7	2.4
KO 6 h	1.1	3.9	3.4
H2O2 3 h	1.8	0	0
H2O2 6 h	1.6	0.3	0

P+I 3 h	21	20	18
P+I 6 h	71	75	69

None 3 h	0	0.3	0
None 6 h	0	0	0.5
IgE 3 h	60	42	31
IgE 6 h	70	60	81

Values for Fig 8B

Sample	Sneezing times/5 min			
No sensitization	2	0	1	0
Sensitization				
None	34	51	36	48
WT	2	18	23	21
KO	43	38	43	39
H2O2	27	15	11	18

Values for Fig 8C

Sample	Scraping times/5 min			
No sensitization	15	14	8	12
Sensitization				
None	48	60	68	37
WT	15	9	26	27
KO	55	34	47	46
H2O2	26	29	29	20
